# Board of Dental Examiners

**Published:** 1878-01

**Authors:** 


### BOARD OF DENTAL EXAMINERS.

The Annual Meeting of the Ohio State Board of Dental Examiners, met in Columbus, December 5, 1877, when the following persons presented themselves for examination, viz: Isaac L. Mitchel, London, Madison Co. Burton R. Shipp, Hillsboro, Highland Co. William H. Sillito, Xenia, Green Co., all having passed a satisfactory examination, received certificates to that effect. The standard of these examinations is being gradually raised, and those who supply, sustain themselves quite as well, if not better than many of those who have gone before them. The time of service of two members, viz: Drs. Horton and Taft, having expired, they were again elected for three years.

